# Workers’ emotional exhaustion and mental well-being over the COVID-19 pandemic: a Dynamic Structural Equation Modeling (DSEM) approach

**DOI:** 10.3389/fpsyg.2023.1222845

**Published:** 2023-10-05

**Authors:** Enrico Perinelli, Michela Vignoli, Friedrich Kröner, Andreas Müller, Melanie Genrich, Franco Fraccaroli

**Affiliations:** ^1^Department of Psychology and Cognitive Science, University of Trento, Rovereto, Italy; ^2^Institute of Psychology, Work & Organizational Psychology, University of Duisburg-Essen, Essen, Germany

**Keywords:** Dynamic Structural Equation Modeling (DSEM), multilevel models, emotional exhaustion, mental well-being, occupational health

## Abstract

The COVID-19 pandemic has presented significant challenges to the workforce, particularly concerning emotional and mental well-being. Given the prolonged periods of work-related stress, unexpected organizational changes, and uncertainties about work faced during the pandemic, it becomes imperative to study occupational health constructs under a dynamic methodological perspective, to understand their stable and unstable characteristics better. In this study, drawing on the Dynamic Structural Equation Modeling (DSEM) framework, we used a combination of multilevel AR(1) models, Residual-DSEM (RDSEM), multilevel bivariate VAR(1) models, and multilevel location-scale models to investigate the autoregression, trend, and (residual) cross-lagged relationships between emotional exhaustion (EmEx) and mental well-being (MWB) over the COVID-19 pandemic. Data were collected weekly on 533 workers from Germany (91.18%) and Italy (8.82%) who completed a self-reported battery (total number of observations = 3,946). Consistent with our hypotheses, results were as follows: (a) regarding *autoregression*, the autoregressive component for both EmEx and MWB was positive and significant, as well as it was their associated between-level variability; (b) regarding *trend*, over time EmEx significantly increased, while MWB significantly declined, furthermore both changes had a significant between-level variability; (c) regarding the longitudinal bivariate (*cross-lagged*) relationships, EmEx and MWB negatively and significantly affected each other from week to week, furthermore both cross-lagged relationships showed to have significant between-level variance. Overall, our study pointed attention to the vicious cycle between EmEx and MWB, even after controlling for their autoregressive component and trend, and supported the utility of DSEM in occupational health psychology studies.

## Introduction

During the CoronaVirus Disease 19 (COVID-19) pandemic, workers globally experienced significant changes, challenges, and threats to their work sphere. Indeed, research attested that the COVID-19 crisis has intensified issues in several work-related areas, such as work-family interface (Vaziri et al., 2020), job search behavior (McFarland et al., 2020), job engagement (Hu et al., 2020), vocational behavior (Newman et al., 2022), and technostress (in particular in aged population; Nimrod, 2022).

All in all, these findings suggest that, on average, workers’ occupational health and well-being underwent significant changes during the COVID-19 pandemic. However, to our knowledge, no study has empirically tested this assumption using an appropriate methodological framework. Specifically, questions about the direction and variability of this change and the stability of occupational health constructs over a set period (e.g., during the COVID-19 pandemic), necessitate intensive longitudinal data collection and specific multivariate data analysis. Such an approach surpasses the scope of more prevalent cross-sectional or longitudinal designs frequently seen in COVID-19 occupational health studies (e.g., Aymerich et al., 2022).

Indeed, scholars concur that research and practice in industrial and organizational psychology substantially changed after the COVID-19 crisis, as both workers and organizations reframed their approach to phenomena such as (among others) working from home, virtual teamwork, social distancing, stress, job insecurity, unemployment, and career choices (Kniffin et al., 2021; Rudolph et al., 2021). Furthermore, as Shoss (2021) put it, “the COVID-19 pandemic is arguably the most widespread and profound occupational health crisis in modern times” (p. 259) and thus research on occupational health psychology has now the unique opportunity (and responsibility) to “generate ideas that may prove useful for addressing future crises” (p. 259).

Aligned with these observations, in this contribution we underscored the potential of a relatively new methodological framework, namely Dynamic Structural Equation Modeling (DSEM; Asparouhov et al., 2018). We aimed to demonstrate its application in occupational health psychology, emphasizing its utility in unraveling the dynamics of workers’ occupational health variables and their relationship. This approach becomes particularly salient when data is sourced from a period rife with profound changes and challenges, such as the COVID-19 pandemic. In more detail, DSEM integrates Structural Equation Modeling (SEM), time-series analysis, and multilevel modeling into a single comprehensive model (Hamaker et al., 2023). There are various possible specifications of DSEM; nevertheless, its general purpose is the analysis of intensive longitudinal data with the incorporation of autoregressive processes (i.e., how a variable at one time point predicts the same variable at a subsequent time point) and multivariate models (i.e., predicting more variables simultaneously), as well as greater flexibility in the estimation of between- and within-level parameters (e.g., simultaneously estimating and predicting between-level variances of trend in the series, autoregressive effects, and reciprocal effects). Overall, DSEMs are particularly useful when understanding the dynamic interplay between variables over time is relevant. In particular, here we investigated the characteristics of emotional exhaustion, mental well-being, and their relationships in a sample of workers followed across the COVID-19 crisis. In what follows, we describe the application of the DSEM framework to the study of emotional exhaustion and mental well-being, we explained why it is important to study occupational health variables over the COVID-19 pandemic, and we presented in more detail our hypotheses. Finally, more technical details on the DSEM specification we used were presented directly in the Method section.

### The DSEM framework applied to the study of emotional exhaustion and mental well-being over the COVID-19 pandemic

Emotional exhaustion is a core component of burnout. It is characterized by symptoms such as difficulties in recovering energy, mental fatigue, and lack of energy, mainly due to work-related causes (e.g., workload; Maslach et al., 2001). On the other side, mental (or psychological) well-being (or health) is a broad construct that – according to the World Health Organization – is characterized by a state “in which the individual realizes his or her abilities, can cope with the normal stresses of life, can work productively and fruitfully, and can make a contribution to his or her community” (Fusar-Poli et al., 2020, p. 41).

In this contribution, the application of the DSEM framework to the study of emotional exhaustion, mental well-being, and their relationship has a three-fold aim. This three-fold aim is mostly rooted in the importance of properly disentangling variance at both within- and between-level (Hamaker, 2012; Hamaker et al., 2015), as it is of particular interest in recent organizational literature (Podsakoff et al., 2019; McCormick et al., 2020; Zyphur et al., 2020a,b; Hofmans et al., 2021).

First, we aimed to investigate the degree of autoregression over the COVID-19 pandemic of emotional exhaustion and mental well-being. According to research on state–trait models (Steyer et al., 2015), psychological constructs (even those considered essentially stable) have a significant degree of both stability and changeability. This means that taking into account the stability of a construct is the first step for probing its development over time (Prenoveau, 2016; Castro-Alvarez et al., 2022; Stadtbaeumer et al., in press). Thus, we hypothesize that both emotional exhaustion and mental well-being should show a significant size of autoregression (*H1*). Also, we hypothesized that these autoregressive components can have a substantial between-level variability, which means that people may differ in the stability (or *inertia* or *carry-over effect*) of emotional exhaustion and mental well-being (*H1a*). In the framework of DSEM, these hypotheses may be investigated using a multilevel AR(1) model. This model is a combination of time-series analysis, multilevel modeling, and SEM (Hamaker et al., 2023), which allows us to investigate the degree to which a variable at time *t* – 1 affects itself over time (i.e., at time *t*), and also allows to investigate the degree of between-level variance of this parameter (McNeish and Hamaker, 2020).

Second, according to the Conservation of Resources (COR) theory, the challenges posed by the COVID-19 pandemic may have significantly depleted workers’ resources, thus creating feelings of discomfort and exacerbating stress over time (Hobfoll et al., 2018). Hence, we hypothesized a significant mean-level increase in emotional exhaustion and a significant mean-level decrease in mental well-being over the course of the COVID-19 pandemic (*H2*). Also, we hypothesized that this mean-level change may significantly vary at the between-level, which means that people may differ in the way they change over time in the two constructs (*H2a*). In the framework of DSEM, these hypotheses may be investigated using a Residual DSEM (RDSEM) with a linear trend (McNeish and Hamaker, 2020).

Third, after taking into consideration the above unconditional characteristics (i.e., autoregression and trend) and sources of within- vs. between-level variance, we aimed to deepen our understanding of the relationship between emotional exhaustion and mental well-being. Indeed, the relationship between emotional exhaustion and mental well-being is attested to be negative by different researchers (Wang and Li, 2015; Jeon et al., 2018), however, there are no studies investigating the direction of the effect over time, or, simply put, the cross-lagged effect exerted on each other (i.e., the effect exerted by a construct *x* at time *t* – 1 on the construct *y* at time *t*, and vice versa, after controlling for the stability of both). In this sense, there are three scenarios: The first provides that one exerts a significant (and negative) cross-lagged effect on the other, but not vice versa; the second provides that no one exerts a significant cross-lagged effect on the other, thus leaving significant only the autoregressive effects; the third provides that both significantly (and negatively) exert a cross-lagged effect on the other. We hypothesized the third scenario (*H3*) in which there is a mutually significant and negative relationship between emotional exhaustion and mental well-being, given the recent findings on the strict relationship between burnout and clinical/health phenomena (Bianchi et al., 2015, 2019, 2021). Also, we hypothesize that the cross-lagged effect exerted by emotional exhaustion on mental well-being has a significant between-level variance (*H3a*) and that the cross-lagged effect exerted by mental well-being on emotional exhaustion has a significant between-level variance (*H3b*). In the framework of DSEM, these hypotheses may be investigated using a revised and combined version of the RDSEM, multilevel AR(1) model, multilevel bivariate VAR(1) model, and multilevel location-scale model (Asparouhov et al., 2018; Hamaker et al., 2018, 2023; McNeish and Hamaker, 2020; McNeish, 2021).

## The present study

To summarize, to advance literature on occupational health psychology and respond to the previously cited call by Shoss (2021), we adopted a substantive-methodological synergy (Hofmans et al., 2021) to investigate (a) the degree of stability (*autoregression*), (b) the development over time (*trend*), (c) the mutual relationship (*cross-lagged*), and (d) their degree of between-level variability of two widely-studied constructs in occupational health psychology (namely, emotional exhaustion and mental well-being) using one of the most advanced latent variable framework available in psychological methods literature, that is DSEM (Asparouhov et al., 2018). Also, differently from the previous application of (R)DSEM (e.g., Hamaker et al., 2018), we provided a parametrization in which autoregression, trends, and cross-lagged relationships are estimated in a unique multivariate model.

## Method

### Participants and procedure

Participants were recruited through various methods, including social media outreach, survey dissemination, and snowball sampling technique. The aim of the project (titled “*Working in Time of Crisis*”) was to assess and study several work-related variables during the COVID-19 pandemic. The study received a positive evaluation from the Ethics Committee of the Institute of Psychology of the University of Duisburg-Essen. All the self-reports used in the survey were gathered from scales widely validated in the international literature (such as the *Burnout Assessment Tool* and the *5-item World Health Organization Well-Being Index* for measuring emotional exhaustion and mental well-being, respectively). The survey took place approximately from the end of March 2020 to May 2021. The research design adopted was that of intensive studies: Each week the subjects who voluntarily took part in the study received an email containing a link that directed to the compilation of the battery. After reading the information about the study and consenting *via* a confirmation form, participants could register using their email address. A link to the survey was then sent to their email. The survey began by collecting demographic and job-related details. Subsequent sections delved into their working conditions, their experiences amid the COVID-19 situation, and their overall well-being. Updates were conducted weekly. At the start of each survey, participants were queried about their work in the preceding week and if any work-related changes had occurred. If there were no job-related alterations, the section surveying working conditions was skipped. If participants missed surveys, reminders were sporadically sent (a maximum of five times). In all invitations and reminders, the option to withdraw from the study was consistently presented.

At the end of the survey, data was collected on 753 workers. However, in subsequent data analysis, we filtered the dataset excluding those who filled less than three waves (i.e., we removed 125 subjects who filled one wave and 94 subjects who filled two waves) and two participants who declared not to work in Germany or Italy. Thus, the final sample consisted of 533 subjects. The count of participants by waves completed is reported in Table 1. The total number of observations is 3,946. The first collection is dated March 24, 2020, while the last is dated March 23, 2021. Regarding nationality, 486 (91.18%) were German while 47 (8.82%) were Italian. Regarding gender, 382 (71.67%) were females and 151 (28.33%) were males. Age ranged from 18 to 70 (*M* = 41.09, *SD* = 12.63), and tenure in the current work role ranged from 0 to 44 years (*M* = 8.69, *SD* = 9.61).

**TABLE 1:** Number of participants by waves completed.

Waves completed	Frequency (*n*)
3	44
4	46
5	52
6	48
7	53
8	47
9	97
10	144
11	2

### Measures

#### Emotional exhaustion

Emotional exhaustion was assessed using three items gathered from the *Burnout Assessment Tool* (BAT; Schaufeli et al., 2020). The introduction to the scale reads “*The following statements are related to your work condition and how you experience this condition. Please state how often each statement applies to you*.” The three items used were “*At work, I feel mentally exhausted*,” “*After a day at work, I find it hard to recover my energy*,” and “*When I get up in the morning, I lack the energy to start a new day at work*.” Items were rated by participants using a 5-point Likert scale ranging from 1 (“*Never*”) to 5 (“*Always*”).

#### Mental well-being

Mental well-being was assessed using the *5-item World Health Organization Well-Being Index* (WHO-5) (World Health Organization, 1998). The WHO-5 is among the most widely used tools assessing subjective psychological well-being and it is translated in more than 30 countries (see Topp et al., 2015). The introduction to the scale reads “*Please indicate for each of the five statements which is the closest one to how you have been feeling over the last week. Notice that higher numbers mean better well-being. Over the last week…*.” The five items were “*I have felt cheerful and in good spirits*,” “*I have felt calm and relaxed*,” “*I have felt active and vigorous*,” “*I woke up feeling fresh and rested*,” and “*My daily life has been filled with things that interest me*.” Items were rated by participants using a 6-point Likert scale ranging from 0 (“*At no time*”) to 5 (“*All of the time*”).

### Data analytic strategy

Data wrangling and reliability analysis were conducted using the statistical open-source software R (Version 4.2.1; R Core Team, 2022). First, the time variable was mutated to have the first occasion equal to zero. Second, we built composite scores for emotional exhaustion and mental well-being by averaging their items, to have a single composite score for each construct. Third, we used the multilevel.reliability function from the psych package (Revelle, 2022) for computing two commonly used reliability indices for multi-item instruments assessed in intensive longitudinal data (Shrout and Lane, 2012; Bolger and Laurenceau, 2013; Revelle and Wilt, 2019): The RkF, which assesses reliability at the between-level, and the reliability of change (Rc), which assesses reliability at the within-level.

Data analyses were conducted with M*plus* Version 8.4 (Muthén and Muthén, 1998–2017).

To test our hypotheses, we used a series of competing Dynamic Structural Equation Models (DSEM). In accordance with methodological literature on DSEM (Asparouhov et al., 2018; Hamaker et al., 2018, 2023; Asparouhov and Muthén, 2020; McNeish and Hamaker, 2020; Wang and Wang, 2020; Geiser, 2021; Zhou et al., 2021), in all models (a) we used a Bayesian estimation method, given that DSEM cannot be estimated with Maximum Likelihood (which is the standard estimation method for SEM),^1^ (b) each variable was latent group-mean centered (see also Lüdtke et al., 2008), (c) the Deviance Information Criterion (DIC) was used to compare competing models, preferring the model with lowest DIC. In particular, the DIC is used to compare the significance of the between-level variance, given that Bayesian credible intervals cannot return the value of 0 for a variance (see McNeish and Hamaker, 2020). We performed three types of models, that were described in more detail below.

#### Autoregression and associated between-level variability (M1 and M1a)

In the first step, we estimated a Multilevel AR(1) Model (see McNeish and Hamaker, 2020). It was performed to estimate the autoregression of emotional exhaustion and mental well-being, as well as its person-level variability. In this model (M1), at the within-level we estimated the effect exerted by the variable at *t* – 1 on the variable at the subsequent time-point. This parameter ($\varphi_{i}$) as well as the intercept ($\alpha_{i}$) were allowed to vary at the between-level part of the model, so that it was possible to estimate the mean ($\gamma_{00}$) and the variance ($\tau_{00}$) of the intercept, and the mean ($\gamma_{10}$) and the variance ($\tau_{11}$) of the slope. More formally, if we label emotional exhaustion or mental well-being with *y*, time with *t*, the individual with *i*, and the latent group-mean centering with the superscript *c*, then this model is represented by the following equations:


$$y_{\mathrm{ti}}=\alpha_{i}+\varphi_{i}y_{\left(t-1\right)i}^{c}+\varepsilon_{\mathrm{ti}}$$

$$\alpha_{i}=\gamma_{00}+\mu_{0i}$$

$$\varphi_{i}=\gamma_{10}+\mu_{1i}$$

Where $\varepsilon_{\mathrm{ti}}∼N\left(0,\sigma^{2}\right)$, $\mu_{0i}\sim N\left(0,\tau_{00}\right)$, and $\mu_{1i}\sim N\left(0,\tau_{11}\right)$.

In a subsequent version of these models (M1a), we fixed the variance of the autoregression (i.e., $\tau_{11}$) to a close-to-be zero value (0.001), and compared the two models using the DIC. For both emotional exhaustion and mental well-being, we expect the autoregression to be positive and significant (*H1*) and the DIC of M1 to be lower than the DIC of M1a (*H1a*), thus attesting that the variance should be not close to zero.

#### Trend in the series (M2 and M2a)

In the second step, we estimated a Residual DSEM (RDSEM), in which we added the effect of time on emotional exhaustion and mental well-being (M2).

The main aim of this model is to attest whether over time there is a significant increase in emotional exhaustion and a significant decrease in mental well-being, and that this change has a significant between-level variance (*H2* and *H2a*). Furthermore, in this model, the RDSEM specification “allows for modeling the autoregression on the within-level residuals rather than on the variable itself” (McNeish and Hamaker, 2020, p. 628). In this way, even in the presence of a significant mean-level change, the *stationarity* assumption – namely the constant expected value over time, or $[E\left(y_{t}\right)=\mu rsqbr$ – is satisfied. The equations of this model are:


$$y_{\mathrm{ti}}=\alpha_{i}+\beta_{1i}{\text{Time}}_{\mathrm{ti}}+\varepsilon_{\mathrm{ti}}$$

$$\varepsilon_{\mathrm{ti}}=\varphi_{i}\varepsilon_{\left(t-1\right)i}+\delta_{\mathrm{ti}}$$

$$\alpha_{i}=\gamma_{00}+\mu_{0i}$$

$$\varphi_{i}=\gamma_{10}+\mu_{1i}$$

$$\beta_{1i}=\gamma_{20}+\mu_{2i}$$

Where $\delta_{\mathrm{ti}}\sim N\left(0,\sigma^{2}\right)$, $\mu_{0i}\sim N\left(0,\tau_{00}\right)$, $\mu_{1i}\sim N\left(0,\tau_{11}\right)$, and $\mu_{2i}\sim N\left(0,\tau_{22}\right)$. Note that the autoregressive effect is no longer included in the equation concerning $y_{\mathrm{ti}}$, but it is instead included in the equation for $\varepsilon_{\mathrm{ti}}$ (i.e., the residual, thus the name RDSEM). Our hypothesis *H2* for emotional exhaustion is satisfied if we found $\gamma_{20}$ to be positive and significant; our hypothesis *H2* for mental well-being is satisfied if we found $\gamma_{20}$ to be negative and significant; finally our hypothesis *H2a* (for both emotional exhaustion and mental well-being) is satisfied if the model M2 has a lower DIC if compared to the model M2a, in which the variance of the random slope associated to time, $\tau_{22}$, is fixed to be zero.

#### Cross-lagged effects and between-level variability (M3, M3a, and M3b)

**FIGURE 1:**
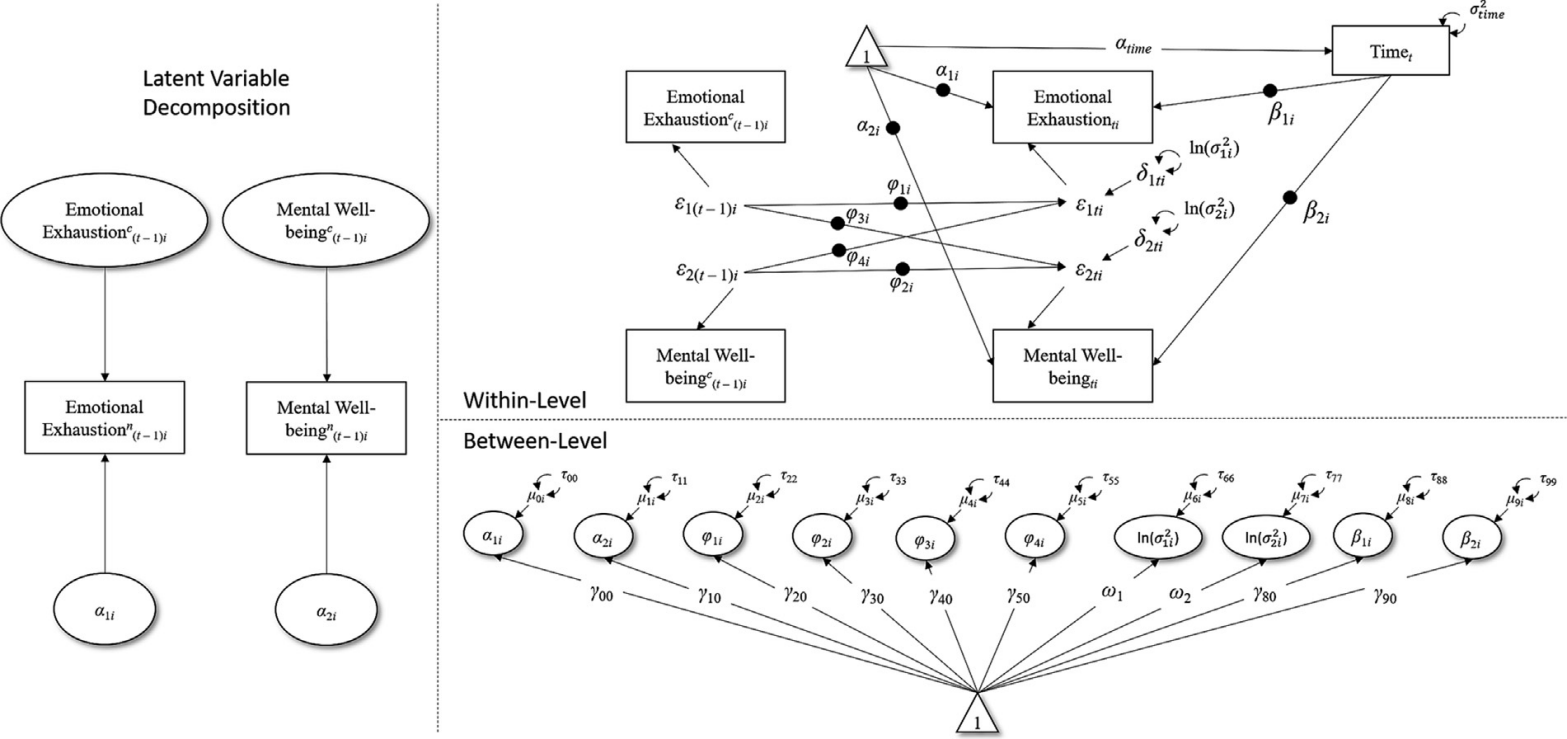
M3: Multilevl Bivariate VAR(1) Model, Superscript *c* = Latent group-mean centered variable; superscript *n* = raw, non-centered version of the variable; subscript *i* = individual; subscript *t* = time. The mean and variance of Time*_t_* at within-level ($\alpha_{\text{time}}$ and $\sigma_{\text{time}}^{2}$, respectively) are estimated because required by M*plus*. Black dots at the center of the arrows represent random parameters.

In the third step, we estimated a multivariate model (M3) which included the specification of (a) a Multilevel VAR(1) Model, (b) a RDSEM with trend in the series, and (c) a multilevel location-scale model (see McNeish and Hamaker, 2020). In more detail, here we specified a model in which emotional exhaustion and mental well-being still have their residual autoregression (that here we label $\varphi_{1i}$ and $\varphi_{2i}$, respectively) and the trend in their series, furthermore we (a) modeled the cross-lagged effect exerted by the *residual* of emotional exhaustion on the *residual* of mental well-being ($\varphi_{3i}$), and computed its between-level mean ($\gamma_{40}$) and variance ($\tau_{44}$); (b) modeled the cross-lagged effect exerted by the *residual* of mental well-being on the *residual* of emotional exhaustion ($\varphi_{4i}$), and computed its between-level mean ($\gamma_{50}$) and variance ($\tau_{55}$); (c) estimated the location-scale model by including the between-person variance of within-person residual variance for both emotional exhaustion ($\sigma_{1i}^{2}$) and mental well-being ($\sigma_{2i}^{2}$); in this specification, the $\sigma^{2}$ is replaced with $\sigma_{i}^{2}$ allowing to obtain a between-person variable represented by the natural logarithm of $\sigma_{i}^{2}$, i.e., $\ln\left(\sigma_{i}^{2}\right)$, with a mean *ω* and a variance *τ*, thus $\sigma_{i}^{2}$ is a function of the exponential of $\omega +\mu_{i}$ namely, $\exp\left(\omega +\mu_{i}\right)$, or $e^{\left(\omega +\mu_{i}\right)}$, where *e* represents Euler’s number (approximatively 2.7182). A graphical representation of the model is provided in Figure 1.

The equations are reported below; note that we used *EmEx* and *MWB* to abbreviate emotional exhaustion and mental well-being, respectively.


$$\text{EmE}x_{\mathrm{ti}}=\alpha_{1i}+\beta_{1i}{\text{Time}}_{\mathrm{ti}}+\varepsilon_{1\mathrm{ti}}$$

$$\text{MW}B_{\mathrm{ti}}=\alpha_{2i}+\beta_{2i}{\text{Time}}_{\mathrm{ti}}+\varepsilon_{2\mathrm{ti}}$$

$$\varepsilon_{1\mathrm{ti}}=\varphi_{1i}\varepsilon_{1\left(t-1\right)i}+\varphi_{4i}\varepsilon_{2\left(t-1\right)i}+\delta_{1\mathrm{ti}},\;\delta_{1\mathrm{ti}}\sim N\left(0,\sigma_{1i}^{2.}\right)\;$$

$$\varepsilon_{2\mathrm{ti}}=\varphi_{2i}\varepsilon_{2\left(t-1\right)i}+\varphi_{3i}\varepsilon_{1\left(t-1\right)i}+\delta_{2\mathrm{ti}},\;\delta_{2\mathrm{ti}}\sim N\left(0,\sigma_{2i}^{2}\right)\;$$

$$\alpha_{1i}=\gamma_{00}+\mu_{0i},\;\mu_{0i}\sim N\left(0,\tau_{00}\right)\;$$

$$\alpha_{2i}=\gamma_{10}+\mu_{1i},\;\mu_{1i}\sim N\left(0,\tau_{11}\right)\;$$

$$\varphi_{1i}=\gamma_{20}+\mu_{2i},\;\mu_{2i}\sim N\left(0,\tau_{22}\right)$$

$$\varphi_{2i}=\gamma_{30}+\mu_{3i},\;\mu_{3i}\sim N\left(0,\tau_{33}\right)$$

$$\varphi_{3i}=\gamma_{40}+\mu_{4i},\;\mu_{4i}\sim N\left(0,\tau_{44}\right)$$

$$\varphi_{4i}=\gamma_{50}+\mu_{5i},\;\mu_{5i}\sim N\left(0,\tau_{55}\right)$$

$$\begin{array}{ccc} \ln\left(\sigma_{1i}^{2}\right) & = & \left(\omega_{0}+\mu_{6i}\right),\;\text{hence}\;\sigma_{1i}^{2}=\exp\left(\omega_{0}+\mu_{6i}\right),\\ & & \mu_{6i}\sim N\left(0,\tau_{66}\right) \end{array}$$

$$\begin{array}{ccc} \ln\left(\sigma_{2i}^{2}\right) & = & \left(\omega_{1}+\mu_{7i}\right),\;\text{hence}\;\sigma_{2i}^{2}=\exp\left(\omega_{1}+\mu_{7i}\right),\;\\ & & \mu_{7i}\sim N\left(0,\tau_{77}\right) \end{array}$$

$$\beta_{1i}=\gamma_{80}+\mu_{8i},\;\mu_{8i}\sim N\left(0,\tau_{88}\right)$$

$$\beta_{2i}=\gamma_{90}+\mu_{9i},\;\mu_{9i}\sim N\left(0,\tau_{99}\right)$$

To support our hypothesis *H3*, we expect that (a) the cross-lagged effect of (residual) emotional exhaustion on (residual) mental well-being ($\gamma_{40}$) is negative and significant as well as (b) the cross-lagged effect of (residual) mental well-being on (residual) emotional exhaustion ($\gamma_{50}$) is negative and significant. To support our hypotheses *H3a* and *H3b* we expect that model M3 has a lower DIC (i.e., it is preferred) if compared to models in which we constrained to a close-to-zero value the variance of the cross-lagged paths, hence we expect that constraining $\tau_{44}$ (M3a) and $\tau_{55}$ (M3b) to 0.001 would worsen the model.

In the Supplementary material, we provide all M*plus* syntaxes, accompanied by annotated comments on the parameters reported above.

## Results

### Reliability analysis

The analysis of multilevel reliability returned satisfactory results. Indeed, at the between-level, the RkF was 0.98 and 0.99 for emotional exhaustion and mental well-being, respectively; at the within-level, the Rc was 0.68 and 0.82 for emotional exhaustion and mental well-being, respectively.

### Autoregression and associated between-level variability (M1 and M1a)

#### Emotional exhaustion

The autoregression effect of emotional exhaustion $\varphi_{i}$ was positive and significant ($\gamma_{10}\;$= 0.32, 95%CI = 0.261, 0.377) and also showed a significant between-level variance ($\tau_{11}\;$= 0.106, 95%CI = 0.08, 0.137). Furthermore, constraining the variance to be close to zero (M1a) increased the DIC of the model (see Table 2). Thus, our hypotheses *H1* and *H1a* were both supported for emotional exhaustion.

**TABLE 2:** Model comparison.

Construct(s)	Model	Description	#Parameters	DIC
EmEx	**M1**	**Unconditional Multilevel AR(1) Model**	**5**	**37829.69**
	M1a	M1 with variance of AR(1) path fixed to a close-to-zero (0.001) value	4	40622.21
	**M2**	**Residual DSEM (RDSEM) with a linear trend**	**9**	**108224.44**
	M2a	RDSEM with a linear trend but its variance fixed to a close-to-zero (0.001) value	8	139776.64
MWB	**M1**	**Unconditional Multilevel AR(1) Model**	**5**	**48175.75**
	M1a	M1 with variance of AR(1) path fixed to a close-to-zero (0.001) value	4	50666.04
	**M2**	**Residual DSEM (RDSEM) with a linear trend**	**9**	**148709.85**
	M2a	RDSEM with a linear trend but its variance fixed to a close-to-zero (0.001) value	8	149756.94
EmEx & MWB	**M3**	**VAR(1) model**	**22**	**174096.25**
	M3a	VAR(1) model with “EmEx → MWB” (*φ*_3_) level-2 variance (*τ*_44_) fixed to a close-to-zero (i.e., 0.001) value	21	174319.45
	M3b	VAR(1) model with “MWB → EmEx” (*φ*_4_) level-2 variance (*τ*_55_) fixed to a close-to-zero (i.e., 0.001) value	21	174955.32

EmEx, emotional exhaustion; MWB, mental well-being; #Parameters, number of estimated parameters; DIC, Deviance Information Criterion. Preferred models are reported in bold.

#### Mental well-being

The autoregression effect of mental well-being $\varphi_{i}$ was positive and significant ($\gamma_{10}\;$= 0.376, 95%CI = 0.31, 0.435) and also showed a significant between-level variance ($\tau_{11}\;$= 0.099, 95%CI = 0.069, 0.135). Furthermore, constraining the variance to be close to zero (M1a) increased the DIC of the model (see Table 2). Thus, our hypotheses *H1* and *H1a* were both supported for mental well-being.

### Trend in the series (M2 and M2a)

#### Emotional exhaustion

Results showed that emotional exhaustion, on average, significantly increased across time ($\gamma_{20}\;$= 0.014, 95%CI = 0.006, 0.027), and also showed a significant between-level variance of this trend ($\tau_{22}\;$= 0.003, 95%CI = 0.002, 0.004). Furthermore, constraining the variance to be close to zero (M2a) increased the DIC of the model (see Table 2). Thus, our hypotheses *H2* and *H2a* were both supported for emotional exhaustion.

#### Mental well-being

Results showed that mental well-being, on average, significantly decreased across time ($\gamma_{20}\;$= −0.014, 95%CI = −0.026, −0.002), and also showed a significant between-level variance of this trend ($\tau_{22}\;$= 0.006, 95%CI = 0.004, 0.008). Furthermore, constraining the variance to be close to zero (M2a) increased the DIC of the model (see Table 2). Thus, our hypotheses *H2* and *H2a* were both supported for mental well-being.

### Cross-lagged effects and between-level variability (M3, M3a, and M3b)

Results from M3 showed that (a) the cross-lagged effect of (residual) emotional exhaustion on (residual) mental well-being was negative and significant ($\gamma_{40}\;$= −0.206, 95%CI = −0.265, −0.136); (b) the cross-lagged effect of (residual) mental well-being on (residual) emotional exhaustion is negative and significant ($\gamma_{50}\;$= −0.198, 95%CI = −0.236, −0.164); (c) that the model M3 had a lower DIC (i.e., it is preferred) if compared to models in which we constrained to a close-to-zero value the variance of the cross-lagged paths: This means that constraining $\tau_{44}$ (M3a) and $\tau_{55}$ (M3b) to 0.001 worsened the model (see Table 2). Thus, *H3*, *H3a*, and *H3b* were all supported. All the parameters estimated in M3 are reported in Table 3.

**TABLE 3:** Estimated parameters for M3.

Level	Parameter	Estimate	95%CI	Significance
Within	*α_time_*	3.601	3.519, 3.688	*
	$\sigma_{\text{time}}^{2}$	6.909	6.617, 7.217	*
Between	$\gamma_{00}$	2.395	2.333, 2.459	*
	$\gamma_{10}$	2.719	2.628, 2.808	*
	$\gamma_{20}$	0.304	0.221, 0.379	*
	$\gamma_{30}$	0.501	0.441, 0.561	*
	$\gamma_{40}$	−0.206	−0.265, −0.136	*
	$\gamma_{50}$	−0.198	−0.236, −0.164	*
	$\gamma_{90}$	−0.011	−0.022, 0.001	n.s.
	$\gamma_{80}$	0.012	0.004, 0.021	*
	$\omega_{1}$	−1.858	−1.962, −1.754	*
	$\omega_{2}$	−1.299	−1.395, −1.201	*
	$\tau_{00}$	0.262	0.206, 0.324	*
	$\tau_{11}$	0.502	0.4, 0.616	*
	$\tau_{22}$	0.072	0.046, 0.106	*
	$\tau_{33}$	0.043	0.027, 0.064	*
	$\tau_{44}$	0.018	0.001, 0.059	*
	$\tau_{55}$	0.020	0.008, 0.037	*
	$\tau_{99}$	0.005	0.004, 0.008	*
	$\tau_{88}$	0.003	0.002, 0.005	*
	$\tau_{66}$	0.495	0.371, 0.647	*
	$\tau_{77}$	0.602	0.468, 0.761	*

CI, (Bayesian) credible intervals; *, significant; n.s., not significant.

In Table 4, we reported the explained variance (expressed as the within-level *R*^2^ averaged across clusters) for emotional exhaustion and mental well-being for each model (i.e., M1, M2, and M3).

**TABLE 4:** Explained variance: within-level R-squares averaged across clusters.

Model	Emotional exhaustion		Mental well-being	
	Estimate	95%CI	Estimate	95%CI
M1	0.206	0.178, 0.233	0.236	0.207, 0.266
M2	0.257	0.141, 0.288	0.297	0.267, 0.331
M3	0.406	0.370, 0.442	0.440	0.394, 0.483

## Discussion

The present contribution applied the DSEM framework to the study of some dynamic characteristics regarding emotional exhaustion, mental well-being, and their relationship over the COVID-19 pandemic. In particular, this contribution investigated (1) autoregression, (2) trend in the series, and (3) cross-lagged relationships for emotional exhaustion and mental well-being, along with their associated between-level variability. In what follows we explained in more detail our findings.

Firstly, according to our first group of hypotheses (*H1* and *H1a*), we found that the autoregression component was significant for both constructs, thus highlighting a certain degree of stability of the constructs. While this finding is not new when we consider all the body of research about Latent State–Trait models and theories (Steyer et al., 2015; Geiser, 2021), it further renews the importance of taking into account autoregression coefficients when studying the relationship of a variable with other ones. This is particularly true in occupational health fields, in which the researcher attempts to find relevant predictors of a phenomenon (e.g., burnout), to enhance the quality of interventions. Yet, when autoregression is not included, estimates may be severely overestimated, given that part of the variance of the outcome could be explained by the stability of the construct (and thus by the construct itself) rather than by other variables. Also, we found that the autoregression path of both variables has a significant between-level variance. This means that the stability (or *inertia*, or *carry-over effect*) of emotional exhaustion and mental well-being may change according to some individual’s characteristics; this finding may open interesting issues for future research, such as testing the between-level variability in the stability of other constructs relevant to occupation health, and (most importantly) to find the antecedents and consequences of the degree of stability. From a practical standpoint, people who have a lower degree of stability in emotional exhaustion may have a greater possibility to “break” their burnout symptomatology after organizational or group interventions, while people with a higher level of stability in emotional exhaustion may receive more benefits if the intervention is focused on those personality dispositions that maintain the symptomatology (in this regard, see literature on personality trait change; Roberts et al., 2017; Bleidorn et al., 2019; Wagner et al., 2020). Hence, recognizing the between-level variance in autoregression paths for both emotional exhaustion and mental well-being underscores the need for personalized interventions.

Secondly, according to our second group of hypotheses (*H2* and *H2a*), we supported that over the COVID-19 crisis (a) emotional exhaustion, on average, significantly increased; (b) mental well-being, on average, significantly decreased, and (c) that both those mean-level changes significantly varied across individuals. Thus, the DSEM framework allowed us to properly disentangle the autoregressive effect from the trend, hence obtaining a clearer picture of the development of emotional exhaustion and mental well-being over the COVID-19 pandemic. Again, discovering that individuals vary in their patterns of change in emotional exhaustion and mental well-being could uncover intriguing avenues for future applied research. Indeed, from a practical standpoint, looking for predictors and outcomes of between-level change in occupational health variables (such as the two we explored) may help in better clarifying which constructs may contrast (or enhance) the development of negative (or positive) phenomena. Also, this finding emphasized the need to monitor and assess trends for regular health assessments in workplaces. This can help in early identification and timely interventions, potentially reducing the adverse impacts on employees. Indeed, monitoring trends may reveal periods characterized by collective or individual heightened levels of emotional exhaustion, that thus might be followed by recovery activities and/or recovery experiences (Sonnentag et al., 2022).

Thirdly, while the above findings referred to the unconditional analyses (i.e., we analyzed the two constructs separately), at the conditional (or bivariate) level we parametrize a RDSEM – in more detail a Multilevel Bivariate VAR(1) Model – that allowed to explore the reciprocal longitudinal effect exerted by the two investigated constructs (i.e., emotional exhaustion and mental well-being), after taking into account their degree of autoregression and trend. According to our third group of hypotheses (*H3, H3a*, and *H3b*), we found support for the strict relationship between burnout and clinical phenomena (Bianchi et al., 2015, 2019, 2021). Indeed, despite a few portions of variance remain to be explained after controlling for autoregression and trend, the week-by-week effect exerted by emotional exhaustion on mental well-being, as well as the week-by-week effect exerted by mental well-being on emotional exhaustion, proved to be negative and significantly different from zero. Also, both cross-lagged relationships had a significant between-level of variance. Again, from a practical perspective, this between-level finding may open new avenues of research in occupational health psychology, as future studies may investigate the degree of between-level variance in other cross-lagged relationships, as well as probing which individual-level factors may affect the strengths of these cross-lagged relationships. Regarding our study variables, the negative reciprocal effects between emotional exhaustion and mental well-being underscore the need for integrated workplace programs. Such programs should address both emotional exhaustion and mental well-being simultaneously. For example, employee assistance programs could offer counseling services for mental well-being alongside training to manage burnout. This holistic approach reduces the risk of ineffectiveness arising from focusing solely on one aspect, be it emotional exhaustion or mental well-being.

### Limitations and future directions

Although the several strengths of this contribution, such as the use of intensive longitudinal data, the use of a novel analytical framework (DSEM), a new specification of RDSEM that allowed us to test our specific hypotheses, and the sufficiently high sample size (if we consider the intensive research design adopted), several limitations should be acknowledged.

First, data analytic strategies related to DSEM are still in early development, and thus we do not know if, in the immediate future, some procedures (such as the model comparison using DIC) may change. Also, the Bayesian estimation method applied to SEM requires some decisions that are not well established (e.g., the choice of the “thinning,” the number of iterations, the number of Markov chains, etc.; see for example, Hamaker et al., 2018; Depaoli, 2021; Geiser, 2021).

Second, we exclusively used self-reported data, hence future studies may adopt other (objective or other reported) measurements of emotional exhaustion and mental well-being.

Third, given the computational demands of the models (all models were run on a desktop computer booked on purpose to the IT office of the Department of Psychology and Cognitive Science at the University of Trento, and the elapsed time was about 6 h) we did not explore either predictors or outcomes or even simple correlations at the between-level; thus, these relations may be investigated in future studies.

Fourth, we collected data exclusively from countries where our research teams were based, namely Germany and Italy, without any other criteria guiding country selection. Also, we concluded data collection in May 2021 due to a notable decline in participant responses to our survey.

Fifth, gender differences were not investigated due to the unbalanced sample (females and males were, respectively, 71.67% and 28.33%) as well as for the Third limitation reported in this paragraph; however, in future studies, our approach allows using gender as a between-level variable for testing hypotheses regarding its effect on the various random slopes and random intercepts (presented in details in this paper) that can be estimated using DSEMs.

Sixth, our DSEMs did not utilize measurement models; instead, emotional exhaustion and mental well-being were gauged by averaging their respective items. Future research might consider incorporating latent variables to (a) control for measurement error and (b) employ measurement invariance routines to verify if scales remain invariant across both time and individuals (Schuurman and Hamaker, 2019; McNeish et al., 2021).

## Conclusion

In conclusion, this contribution aimed to show the applicability of the DSEM framework to unravel autoregression, trend, cross-lagged relations, and their associated between-level variance in two of the most studied constructs in the field of occupational health psychology, namely emotional exhaustion and mental well-being. We found support for the importance of studying relationships among occupational health constructs employing the DSEM framework, in particular during a challenging (and full of changes) period such as the COVID-19 pandemic (Shoss, 2021). Indeed, (a) we found a significant increase and decline in emotional exhaustion and mental well-being, respectively; (b) we found the negative week-to-week relationship between emotional exhaustion and mental well-being to be significant even after taking into account autoregressions and trends; (c) we found that autoregressions, trends, and cross-lagged relationships all had a significant degree of between-level variance, and discussed the consequences; and finally, (d) we provided a new specification of RDSEM that offered the possibility to explore cross-lagged relationships. Furthermore, while the primary focus of this contribution is methodological, we highlighted how results have potential applications for workplace interventions and practical strategies. Hence, we hope that this substantive-methodological contribution may advance and stimulate future similar applications in the field of occupational health psychology.

## Data availability statement

The original contributions presented in the study are included in the article/Supplementary material, further inquiries can be directed to the corresponding author.

## Ethics statement

All procedures involving research study participants were approved by the ethics committee of the Institute of Psychology of the University of Duisburg-Essen. The studies were conducted in accordance with the local legislation and institutional requirements. The participants provided their written informed consent to participate in this study.

## Author contributions

FK and AM contributed to conception and design of the research project. EP conceptualized and drafted the manuscript and conducted all the statistical analyses. MV, FK, AM, and MG collected the data. All authors were involved in the original project, revised, contributed, and approved the submitted version.

## Footnotes

^1^More information on Bayesian analysis specifications (e.g., number of iterations, thinning, number of chains) can be found in M*plus* syntaxes (under the command ANALYSIS) reported in Supplementary material.
