# Supplementary material for: Workers’ emotional exhaustion and mental well-being over the COVID-19 pandemic: a Dynamic Structural Equation Modeling (DSEM) approach
Source: Front Psychol. 2023 Oct 5;14:1222845. doi: 10.3389/fpsyg.2023.1222845 (PMC10585024; doi:10.3389/fpsyg.2023.1222845)
Supplement: Supplementary file 1 [file Data_Sheet_1.DOCX]

**Supplementary Material**

M*plus* syntaxes for models reported in Table 2.

Note that TINTERVAL option is “used to create a new time variable and insert missing data records when data are misaligned with respect to time” (Muthén, Asparouhov, & Hamaker, 2017, slide#33 available at <https://www.statmodel.com/download/2017MarchPSMGtalkMuthen2.pdf>; see also McNeish & Hamaker, 2020, for more information about TINTERVAL option). As it can be seen from the M*plus* syntaxes reported below, we specified TINTERVAL = day_var(7); where day_var is a variable in which the date of questionnaire administration (stored in real_date) was mutated to the number of days from the first administration (which took place on March 24, 2020): An extract of R syntax to create day_var is: data_covid_ita_deu %<>% mutate(day_var = as.numeric(real_date - as.Date("2020-03-24")). Then, the value of (7) was chosen because it represents 1 week, which was the expected time-lag between one questionnaire administration and the subsequent. To help the correct interpretation of day_var, below we reported a screenshot of the first five rows of the dataset:


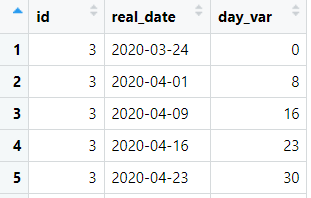


Finally, regarding the match between the name of the variables used in the subsequent syntaxes and the names used in the article (e.g., see Figure 1):

- burnout refers to Emotional Exhaustion
- who refers to Mental Well-being
- sing_obs refers to Time; it is a variable indicating the position of each observation (0, 1, 2, 3, 4… for each id value) according to day_var. It is created because it is not possible to use a variable both in the TINTERVAL and in the MODEL. However, notice that the correlation between sing_obs and day_var is .87.

**Construct = Emotional Exhaustion; Model = M1**

TITLE:

Unconditional Multilevel AR(1) Model

DATA:

file is covid.dat;

VARIABLE:

NAMES =

id day_var sing_obs n_of_obs nation exp_years

age gender kids language educ

bat_EH1 bat_EH3 bat_EH5 burnout

who_who1 who_who2 who_who3 who_who4 who_who5 who;

CLUSTER = id;

WITHIN = ;

BETWEEN = ;

LAGGED = burnout(1); !Create Lag-1

TINTERVAL = day_var(7);

usevariable are

burnout;

MISSING=.;

ANALYSIS:

type is twolevel random; !Two-Level Model with paths that are latent variables;

estimator=bayes; !Lagged variables can only be estimated with Bayes in Mplus;

processors=2; ! Use two processors

bseed = 1234; ! seed for MCMC random number generation

biterations = (5000);

! specifies the minimum number of iterations

! for each MCMC chain when the

! potential scale reduction (PSR)

! convergence criterion (Gelman & Rubin, 1992) is used.

! The first half of the iterations is always used as burn-in

point = median; !Estimating the median is the default for Mplus

chains = 2; !number of MCMC chains (2 is the default);

bconvergence = .05; !MCMC convergence criterion using Gelman-Rubin PSR

! (.05 is the default)

thin = 5; !use estimate from every 5th iteration

MODEL:

%within%

phi | burnout ON burnout&1; ! y is regressed on Lag-1 y, the slope is latent;

burnout; !within-level variance, sigmaˆ2;

%BETWEEN%

[burnout]; !mean intercept, gamma_00;

[phi]; !mean of autoregressive slope, gamma_10;

burnout; !intercept variance, tau_00;

phi; !autoregressive slope variance, tau_11;

OUTPUT:

tech1 tech3 tech8 stdyx stand(cluster) cinterval;

PLOT:

TYPE = PLOT3;

**Construct = Emotional Exhaustion; Model = M1a**

TITLE:

M1 with variance of AR(1) path fixed to a close-to-zero (0.001) value

DATA:

file is covid.dat;

VARIABLE:

NAMES =

id day_var sing_obs n_of_obs nation exp_years

age gender kids language educ

bat_EH1 bat_EH3 bat_EH5 burnout

who_who1 who_who2 who_who3 who_who4 who_who5 who;

CLUSTER = id;

WITHIN = ;

BETWEEN = ;

LAGGED = burnout(1); !Create Lag-1;

TINTERVAL = day_var(7);

usevariable are

burnout;

MISSING=.;

ANALYSIS:

type is twolevel random; !Two-Level Model with paths that are latent variables;

estimator=bayes; !Lagged variables can only be estimated with Bayes in Mplus;

processors=2; ! Use two processors

bseed = 1234; ! seed for MCMC random number generation

biterations = (5000);

! specifies the minimum number of iterations

! for each MCMC chain when the

! potential scale reduction (PSR)

! convergence criterion (Gelman & Rubin, 1992) is used.

! The first half of the iterations is always used as burn-in

point = median; !Estimating the median is the default for Mplus

chains = 2; !number of MCMC chains (2 is the default);

bconvergence = .05; !MCMC convergence criterion using Gelman-Rubin PSR

! (.05 is the default)

thin = 5; !use estimate from every 5th iteration

MODEL:

%WITHIN%

phi | burnout ON burnout&1; ! y is regressed on Lag-1 y, the slope is latent;

burnout; !within-level variance, sigmaˆ2;

%BETWEEN%

[burnout]; !mean intercept, gamma_00;

[phi]; !mean of autoregressive slope, gamma_10;

burnout; !intercept variance, tau_00;

phi@0.001; !autoregressive slope variance, tau_11;

!fixed to be zero;

OUTPUT:

tech1 tech3 tech8 stdyx stand(cluster) cinterval;

PLOT:

TYPE = PLOT3;

**Construct = Emotional Exhaustion; Model = M2**

TITLE:

Residual DSEM (RDSEM) with a linear trend

DATA:

file is covid.dat;

VARIABLE:

NAMES =

id day_var sing_obs n_of_obs nation exp_years

age gender kids language educ

bat_EH1 bat_EH3 bat_EH5 burnout

who_who1 who_who2 who_who3 who_who4 who_who5 who;

CLUSTER = id;

WITHIN = sing_obs; ! add time at the within level

BETWEEN = ;

LAGGED = burnout(1); !Create Lag-1;

TINTERVAL = day_var(7);

usevariable are

sing_obs

burnout;

MISSING=.;

ANALYSIS:

type is twolevel random; !Two-Level Model with paths that are latent variables;

estimator=bayes; !Lagged variables can only be estimated with Bayes in Mplus;

processors=2; ! Use two processors

bseed = 1234; ! seed for MCMC random number generation

biterations = (1700);

! specifies the minimum number of iterations

! for each MCMC chain when the

! potential scale reduction (PSR)

! convergence criterion (Gelman & Rubin, 1992) is used.

! The first half of the iterations is always used as burn-in

point = median; !Estimating the median is the default for Mplus

chains = 2; !number of MCMC chains (2 is the default);

bconvergence = .05; !MCMC convergence criterion using Gelman-Rubin PSR

! (.05 is the default)

thin = 5; !use estimate from every 5th iteration

MODEL:

%WITHIN%

PHI | burnout^ on burnout^1;

! The “ ^ “ symbol is shorthand for residual;

! The residual of y is regressed on Lag-1 residual of y,

! the slope is latent;

TREND | burnout on sing_obs;

! y is regressed on sing_obs to capture the trend over time,

! the slope is latent;

burnout;

!within-level variance, sigma^2;

sing_obs;

%BETWEEN%

[burnout]; !mean intercept, gamma_00;

[PHI]; !mean of autoregressive slope,gamma_10;

[TREND]; !mean of the linear trend slope, gamma_20;

burnout; !intercept variance, tau_00;

PHI; !autoregressive slope variance, tau_11;

TREND; !linear trend slope variance, tau_22;

OUTPUT:

tech1 tech3 tech8 stdyx stand(cluster) cinterval;

PLOT:

TYPE = PLOT3;

**Construct = Emotional Exhaustion; Model = M2a**

TITLE:

RDSEM with a linear trend but its variance fixed to a close-to-zero (0.001) value

DATA:

file is covid.dat;

VARIABLE:

NAMES =

id day_var sing_obs n_of_obs nation exp_years

age gender kids language educ

bat_EH1 bat_EH3 bat_EH5 burnout

who_who1 who_who2 who_who3 who_who4 who_who5 who;

CLUSTER = id;

WITHIN = sing_obs; ! add time at the within level

BETWEEN = ;

LAGGED = burnout(1); !Create Lag-1;

TINTERVAL = day_var(7);

usevariable are

sing_obs

burnout;

MISSING=.;

ANALYSIS:

type is twolevel random; !Two-Level Model with paths that are latent variables;

estimator=bayes; !Lagged variables can only be estimated with Bayes in Mplus;

processors=2; ! Use two processors

bseed = 1234; ! seed for MCMC random number generation

biterations = (5000);

! specifies the minimum number of iterations

! for each MCMC chain when the

! potential scale reduction (PSR)

! convergence criterion (Gelman & Rubin, 1992) is used.

! The first half of the iterations is always used as burn-in

point = median; !Estimating the median is the default for Mplus

chains = 2; !number of MCMC chains (2 is the default);

bconvergence = .05; !MCMC convergence criterion using Gelman-Rubin PSR

! (.05 is the default)

thin = 5; !use estimate from every 5th iteration

MODEL:

%WITHIN%

PHI | burnout^ on burnout^1;

! The “ ^ “ symbol is shorthand for residual;

! The residual of y is regressed on Lag-1 residual of y,

! the slope is latent;

TREND | burnout on sing_obs;

! y is regressed on sing_obs to capture the trend over time,

! the slope is latent;

burnout;

!within-level variance, sigma^2;

sing_obs;

%BETWEEN%

[burnout]; !mean intercept, gamma_00;

[PHI]; !mean of autoregressive slope,gamma_10;

[TREND]; !mean of the linear trend slope, gamma_20;

burnout; !intercept variance, tau_00;

PHI; !autoregressive slope variance, tau_11;

TREND@0.001; !linear trend slope variance, tau_22;

! Fixed to be zero;

OUTPUT:

tech1 tech3 tech8 stdyx stand(cluster) cinterval;

PLOT:

TYPE = PLOT3;

**Construct = Mental Well-being; Model = M1**

TITLE:

Unconditional Multilevel AR(1) Model

DATA:

file is covid.dat;

VARIABLE:

NAMES =

id day_var sing_obs n_of_obs nation exp_years

age gender kids language educ

bat_EH1 bat_EH3 bat_EH5 burnout

who_who1 who_who2 who_who3 who_who4 who_who5 who;

CLUSTER = id;

WITHIN = ;

BETWEEN = ;

LAGGED = who(1); !Create Lag-1

TINTERVAL = day_var(7);

usevariable are

who;

MISSING=.;

ANALYSIS:

type is twolevel random; !Two-Level Model with paths that are latent variables;

estimator=bayes; !Lagged variables can only be estimated with Bayes in Mplus;

processors=2; ! Use two processors

bseed = 1234; ! seed for MCMC random number generation

biterations = (5000);

! specifies the minimum number of iterations

! for each MCMC chain when the

! potential scale reduction (PSR)

! convergence criterion (Gelman & Rubin, 1992) is used.

! The first half of the iterations is always used as burn-in

point = median; !Estimating the median is the default for Mplus

chains = 2; !number of MCMC chains (2 is the default);

bconvergence = .05; !MCMC convergence criterion using Gelman-Rubin PSR

! (.05 is the default)

thin = 5; !use estimate from every 5th iteration

MODEL:

%within%

phi | who ON who&1; ! y is regressed on Lag-1 y, the slope is latent;

who; !within-level variance, sigmaˆ2;

%BETWEEN%

[who]; !mean intercept, gamma_00;

[phi]; !mean of autoregressive slope, gamma_10;

who; !intercept variance, tau_00;

phi; !autoregressive slope variance, tau_11;

OUTPUT:

tech1 tech3 tech8 stdyx stand(cluster) cinterval;

PLOT:

TYPE = PLOT3;

**Construct = Mental Well-being; Model = M1a**

TITLE:

M1 with variance of AR(1) path fixed to a close-to-zero (0.001) value

DATA:

file is covid.dat;

VARIABLE:

NAMES =

id day_var sing_obs n_of_obs nation exp_years

age gender kids language educ

bat_EH1 bat_EH3 bat_EH5 burnout

who_who1 who_who2 who_who3 who_who4 who_who5 who;

CLUSTER = id;

WITHIN = ;

BETWEEN = ;

LAGGED = who(1); !Create Lag-1;

TINTERVAL = day_var(7);

usevariable are

who;

MISSING=.;

ANALYSIS:

type is twolevel random; !Two-Level Model with paths that are latent variables;

estimator=bayes; !Lagged variables can only be estimated with Bayes in Mplus;

processors=2; ! Use two processors

bseed = 1234; ! seed for MCMC random number generation

biterations = (5000);

! specifies the minimum number of iterations

! for each MCMC chain when the

! potential scale reduction (PSR)

! convergence criterion (Gelman & Rubin, 1992) is used.

! The first half of the iterations is always used as burn-in

point = median; !Estimating the median is the default for Mplus

chains = 2; !number of MCMC chains (2 is the default);

bconvergence = .05; !MCMC convergence criterion using Gelman-Rubin PSR

! (.05 is the default)

thin = 5; !use estimate from every 5th iteration

MODEL:

%WITHIN%

phi | who ON who&1; ! y is regressed on Lag-1 y, the slope is latent;

who; !within-level variance, sigmaˆ2;

%BETWEEN%

[who]; !mean intercept, gamma_00;

[phi]; !mean of autoregressive slope, gamma_10;

who; !intercept variance, tau_00;

phi@0.001; !autoregressive slope variance, tau_11;

!fixed to be zero;

OUTPUT:

tech1 tech3 tech8 stdyx stand(cluster) cinterval;

PLOT:

TYPE = PLOT3;

**Construct = Mental Well-being; Model = M2**

TITLE:

Residual DSEM (RDSEM) with a linear trend

DATA:

file is covid.dat;

VARIABLE:

NAMES =

id day_var sing_obs n_of_obs nation exp_years

age gender kids language educ

bat_EH1 bat_EH3 bat_EH5 burnout

who_who1 who_who2 who_who3 who_who4 who_who5 who;

CLUSTER = id;

WITHIN = sing_obs; ! add time at the within level

BETWEEN = ;

LAGGED = who(1); !Create Lag-1;

TINTERVAL = day_var(7);

usevariable are

sing_obs

who;

MISSING=.;

ANALYSIS:

type is twolevel random; !Two-Level Model with paths that are latent variables;

estimator=bayes; !Lagged variables can only be estimated with Bayes in Mplus;

processors=2; ! Use two processors

bseed = 1234; ! seed for MCMC random number generation

biterations = (5000);

! specifies the minimum number of iterations

! for each MCMC chain when the

! potential scale reduction (PSR)

! convergence criterion (Gelman & Rubin, 1992) is used.

! The first half of the iterations is always used as burn-in

point = median; !Estimating the median is the default for Mplus

chains = 2; !number of MCMC chains (2 is the default);

bconvergence = .05; !MCMC convergence criterion using Gelman-Rubin PSR

! (.05 is the default)

thin = 5; !use estimate from every 5th iteration

MODEL:

%WITHIN%

PHI | who^ on who^1;

! The “ ^ “ symbol is shorthand for residual;

! The residual of y is regressed on Lag-1 residual of y,

! the slope is latent;

TREND | who on sing_obs;

! y is regressed on sing_obs to capture the trend over time,

! the slope is latent;

who;

!within-level variance, sigma^2;

sing_obs;

%BETWEEN%

[who]; !mean intercept, gamma_00;

[PHI]; !mean of autoregressive slope,gamma_10;

[TREND]; !mean of the linear trend slope, gamma_20;

who; !intercept variance, tau_00;

PHI; !autoregressive slope variance, tau_11;

TREND; !linear trend slope variance, tau_22;

OUTPUT:

tech1 tech3 tech8 stdyx stand(cluster) cinterval;

PLOT:

TYPE = PLOT3;

**Construct = Mental Well-being; Model = M2a**

TITLE:

RDSEM with a linear trend but its variance fixed to a close-to-zero (0.001) value

DATA:

file is covid.dat;

VARIABLE:

NAMES =

id day_var sing_obs n_of_obs nation exp_years

age gender kids language educ

bat_EH1 bat_EH3 bat_EH5 burnout

who_who1 who_who2 who_who3 who_who4 who_who5 who;

CLUSTER = id;

WITHIN = sing_obs; ! add time at the within level

BETWEEN = ;

LAGGED = who(1); !Create Lag-1;

TINTERVAL = day_var(7);

usevariable are

sing_obs

who;

MISSING=.;

ANALYSIS:

type is twolevel random; !Two-Level Model with paths that are latent variables;

estimator=bayes; !Lagged variables can only be estimated with Bayes in Mplus;

processors=2; ! Use two processors

bseed = 1234; ! seed for MCMC random number generation

biterations = (5000);

! specifies the minimum number of iterations

! for each MCMC chain when the

! potential scale reduction (PSR)

! convergence criterion (Gelman & Rubin, 1992) is used.

! The first half of the iterations is always used as burn-in

point = median; !Estimating the median is the default for Mplus

chains = 2; !number of MCMC chains (2 is the default);

bconvergence = .05; !MCMC convergence criterion using Gelman-Rubin PSR

! (.05 is the default)

thin = 5; !use estimate from every 5th iteration

MODEL:

%WITHIN%

PHI | who^ on who^1;

! The “ ^ “ symbol is shorthand for residual;

! The residual of y is regressed on Lag-1 residual of y,

! the slope is latent;

TREND | who on sing_obs;

! y is regressed on sing_obs to capture the trend over time,

! the slope is latent;

who;

!within-level variance, sigma^2;

sing_obs;

%BETWEEN%

[who]; !mean intercept, gamma_00;

[PHI]; !mean of autoregressive slope,gamma_10;

[TREND]; !mean of the linear trend slope, gamma_20;

who; !intercept variance, tau_00;

PHI; !autoregressive slope variance, tau_11;

TREND@0.001; !linear trend slope variance, tau_22;

! Fixed to be zero;

OUTPUT:

tech1 tech3 tech8 stdyx stand(cluster) cinterval;

PLOT:

TYPE = PLOT3;

**Constructs = Emotional Exhaustion & Mental Well-being; Model = M3**

TITLE:

VAR(1) model

! Note that

! - burnout = emotional exhaustion

! - who = mental well-being

DATA:

file is covid.dat;

VARIABLE:

NAMES =

id day_var sing_obs n_of_obs nation exp_years

age gender kids language educ

bat_EH1 bat_EH3 bat_EH5 burnout

who_who1 who_who2 who_who3 who_who4 who_who5 who;

CLUSTER = id;

WITHIN = sing_obs; ! add time at the within level

BETWEEN = ;

LAGGED = who(1) burnout(1); !Create Lag-1 for who and burnout;

TINTERVAL = day_var(7);

usevariable are

sing_obs

burnout

who;

MISSING=.;

ANALYSIS:

type is twolevel random; !Two-Level Model with paths that are latent variables;

estimator=bayes; !Lagged variables can only be estimated with Bayes in Mplus;

processors=2; ! Use two processors

bseed = 1234; ! seed for MCMC random number generation

biterations = (5000);

! specifies the minimum number of iterations

! for each MCMC chain when the

! potential scale reduction (PSR)

! convergence criterion (Gelman & Rubin, 1992) is used.

! The first half of the iterations is always used as burn-in

point = median; !Estimating the median is the default for Mplus

chains = 2; !number of MCMC chains (2 is the default);

bconvergence = .05; !MCMC convergence criterion using Gelman-Rubin PSR

! (.05 is the default)

thin = 5; !use estimate from every 5th iteration

MODEL:

%WITHIN%

! Autoregression with residuals ----------------------------------------

phi1 | burnout^ on burnout^1;

! The “ ^ “ symbol is shorthand for residual;

! The residual of burnout is regressed on Lag-1 residual of burnout,

! the slope is latent;

phi2 | who^ on who^1;

! The “ ^ “ symbol is shorthand for residual;

! The residual of who is regressed on Lag-1 residual of who,

! the slope is latent;

! Cross-lagged ---------------------------------------------------------

phi3 | who^ on burnout^1;!residual of who is regressed on Lag-1 residual of burnout, phi_3i

!the slope is latent;

phi4 |burnout^ on who^1;! residual of burnout is regressed on Lag-1 residual of who, phi_4i

!the slope is latent;

! Trends ----------------------------------------------------------------

trend_wh | who on sing_obs;

! who is regressed on sing_obs to capture the trend over time,

!the slope is latent;

trend_bu | burnout on sing_obs;

! burnout is regressed on sing_obs to capture the trend over time,

!the slope is latent;

! Within-level residual variances ----------------------------------------

logv1 | burnout;!the Within-Level residual variance of burnout is latent,

!sigmaˆ2_1i;

logv2 | who;!the Within-Level residual variance of who is latent,

!sigmaˆ2_2i;

! Compute the variance (and mean, by default) of Time ---------------------

sing_obs; ! to be inserted, otherwise Mplus returns an error

%BETWEEN%

[burnout]; !mean intercept of burnout, gamma_00;

[who]; !mean intercept of who, gamma_10;

[phi1]; !mean of burnout autoregressive slope, gamma_20;

[phi2]; !mean of who autoregressive slope,gamma_30;

[phi3]; !mean of who^ on burnout^1 slope, gamma_40;

[phi4]; !mean of burnout^ on who^1 slope, gamma_50;

[logv1]; !log of the mean of the burnout residual variance, omega_0;

[logv2]; !log of the mean of the who residual variance, omega_1;

[trend_bu];!mean of trend for burnout, gamma_80;

[trend_wh];!mean of trend for who, gamma_90;

burnout; !burnout intercept variance, tau_00;

who; !who intercept variance, tau_11;

phi1; !burnout autoregressive slope variance, tau_22;

phi2; !who autoregressive slope variance, tau_33;

phi3; !who^ on burnout^1 slope variance, tau_44;

phi4; !burnout^ on who^1 slope,tau_55;

logv1; !between-person variance of burnout residual variance, tau_66;

logv2; !between-person variance of who residual variance, tau_77;

trend_bu;!between-person variance of burnout trend, tau_88;

trend_wh;!between-person variance of who trend, tau_99;

OUTPUT:

tech1 tech3 tech8 stdyx stand(cluster) cinterval;

PLOT:

TYPE = PLOT3;

**Constructs = Emotional Exhaustion & Mental Well-being; Model = M3a**

TITLE:

VAR(1) model with "Em Exth -> MWB" (phi3) level-2 variance (tau44)

fixed to a close-to-zero (i.e., 0.001) value

! Note that

! - burnout = emotional exhaustion

! - who = mental well-being

DATA:

file is covid.dat;

VARIABLE:

NAMES =

id day_var sing_obs n_of_obs nation exp_years

age gender kids language educ

bat_EH1 bat_EH3 bat_EH5 burnout

who_who1 who_who2 who_who3 who_who4 who_who5 who;

CLUSTER = id;

WITHIN = sing_obs; ! add time at the within level

BETWEEN = ;

LAGGED = who(1) burnout(1); !Create Lag-1 for who and burnout;

TINTERVAL = day_var(7);

usevariable are

sing_obs

burnout

who;

MISSING=.;

ANALYSIS:

type is twolevel random; !Two-Level Model with paths that are latent variables;

estimator=bayes; !Lagged variables can only be estimated with Bayes in Mplus;

processors=2; ! Use two processors

bseed = 1234; ! seed for MCMC random number generation

biterations = (5000);

! specifies the minimum number of iterations

! for each MCMC chain when the

! potential scale reduction (PSR)

! convergence criterion (Gelman & Rubin, 1992) is used.

! The first half of the iterations is always used as burn-in

point = median; !Estimating the median is the default for Mplus

chains = 2; !number of MCMC chains (2 is the default);

bconvergence = .05; !MCMC convergence criterion using Gelman-Rubin PSR

! (.05 is the default)

thin = 5; !use estimate from every 5th iteration

MODEL:

%WITHIN%

! Autoregression with residuals ----------------------------------------

phi1 | burnout^ on burnout^1;

! The “ ^ “ symbol is shorthand for residual;

! The residual of burnout is regressed on Lag-1 residual of burnout,

! the slope is latent;

phi2 | who^ on who^1;

! The “ ^ “ symbol is shorthand for residual;

! The residual of who is regressed on Lag-1 residual of who,

! the slope is latent;

! Cross-lagged ---------------------------------------------------------

phi3 | who^ on burnout^1;!residual of who is regressed on Lag-1 residual of burnout, phi_3i

!the slope is latent;

phi4 |burnout^ on who^1;! residual of burnout is regressed on Lag-1 residual of who, phi_4i

!the slope is latent;

! Trends ----------------------------------------------------------------

trend_wh | who on sing_obs;

! who is regressed on sing_obs to capture the trend over time,

!the slope is latent;

trend_bu | burnout on sing_obs;

! burnout is regressed on sing_obs to capture the trend over time,

!the slope is latent;

! Within-level residual variances ----------------------------------------

logv1 | burnout;!the Within-Level residual variance of burnout is latent,

!sigmaˆ2_1i;

logv2 | who;!the Within-Level residual variance of who is latent,

!sigmaˆ2_2i;

! Compute the variance (and mean, by default) of Time ---------------------

sing_obs; ! to be inserted, otherwise Mplus returns an error

%BETWEEN%

[burnout]; !mean intercept of burnout, gamma_00;

[who]; !mean intercept of who, gamma_10;

[phi1]; !mean of burnout autoregressive slope, gamma_20;

[phi2]; !mean of who autoregressive slope,gamma_30;

[phi3]; !mean of who^ on burnout^1 slope, gamma_40;

[phi4]; !mean of burnout^ on who^1 slope, gamma_50;

[logv1]; !log of the mean of the burnout residual variance, omega_0;

[logv2]; !log of the mean of the who residual variance, omega_1;

[trend_bu];!mean of trend for burnout, gamma_80;

[trend_wh];!mean of trend for who, gamma_90;

burnout; !burnout intercept variance, tau_00;

who; !who intercept variance, tau_11;

phi1; !burnout autoregressive slope variance, tau_22;

phi2; !who autoregressive slope variance, tau_33;

phi3@0.001; !who^ on burnout^1 slope variance, tau_44;

phi4; !burnout^ on who^1 slope,tau_55;

logv1; !between-person variance of burnout residual variance, tau_66;

logv2; !between-person variance of who residual variance, tau_77;

trend_bu;!between-person variance of burnout trend, tau_88;

trend_wh;!between-person variance of who trend, tau_99;

OUTPUT:

tech1 tech3 tech8 stdyx stand(cluster) cinterval;

PLOT:

TYPE = PLOT3;

**Constructs = Emotional Exhaustion & Mental Well-being; Model = M3b**

TITLE:

VAR(1) model with "MWB -> Em Exth" (phi4) level-2 variance (tau55)

fixed to a close-to-zero (i.e., 0.001) value

! Note that

! - burnout = emotional exhaustion

! - who = mental well-being

DATA:

file is covid.dat;

VARIABLE:

NAMES =

id day_var sing_obs n_of_obs nation exp_years

age gender kids language educ

bat_EH1 bat_EH3 bat_EH5 burnout

who_who1 who_who2 who_who3 who_who4 who_who5 who;

CLUSTER = id;

WITHIN = sing_obs; ! add time at the within level

BETWEEN = ;

LAGGED = who(1) burnout(1); !Create Lag-1 for who and burnout;

TINTERVAL = day_var(7);

usevariable are

sing_obs

burnout

who;

MISSING=.;

ANALYSIS:

type is twolevel random; !Two-Level Model with paths that are latent variables;

estimator=bayes; !Lagged variables can only be estimated with Bayes in Mplus;

processors=2; ! Use two processors

bseed = 1234; ! seed for MCMC random number generation

biterations = (5000);

! specifies the minimum number of iterations

! for each MCMC chain when the

! potential scale reduction (PSR)

! convergence criterion (Gelman & Rubin, 1992) is used.

! The first half of the iterations is always used as burn-in

point = median; !Estimating the median is the default for Mplus

chains = 2; !number of MCMC chains (2 is the default);

bconvergence = .05; !MCMC convergence criterion using Gelman-Rubin PSR

! (.05 is the default)

thin = 5; !use estimate from every 5th iteration

MODEL:

%WITHIN%

! Autoregression with residuals ----------------------------------------

phi1 | burnout^ on burnout^1;

! The “ ^ “ symbol is shorthand for residual;

! The residual of burnout is regressed on Lag-1 residual of burnout,

! the slope is latent;

phi2 | who^ on who^1;

! The “ ^ “ symbol is shorthand for residual;

! The residual of who is regressed on Lag-1 residual of who,

! the slope is latent;

! Cross-lagged ---------------------------------------------------------

phi3 | who^ on burnout^1;!residual of who is regressed on Lag-1 residual of burnout, phi_3i

!the slope is latent;

phi4 |burnout^ on who^1;! residual of burnout is regressed on Lag-1 residual of who, phi_4i

!the slope is latent;

! Trends ----------------------------------------------------------------

trend_wh | who on sing_obs;

! who is regressed on sing_obs to capture the trend over time,

!the slope is latent;

trend_bu | burnout on sing_obs;

! burnout is regressed on sing_obs to capture the trend over time,

!the slope is latent;

! Within-level residual variances ----------------------------------------

logv1 | burnout;!the Within-Level residual variance of burnout is latent,

!sigmaˆ2_1i;

logv2 | who;!the Within-Level residual variance of who is latent,

!sigmaˆ2_2i;

! Compute the variance (and mean, by default) of Time ---------------------

sing_obs; ! to be inserted, otherwise Mplus returns an error

%BETWEEN%

[burnout]; !mean intercept of burnout, gamma_00;

[who]; !mean intercept of who, gamma_10;

[phi1]; !mean of burnout autoregressive slope, gamma_20;

[phi2]; !mean of who autoregressive slope,gamma_30;

[phi3]; !mean of who^ on burnout^1 slope, gamma_40;

[phi4]; !mean of burnout^ on who^1 slope, gamma_50;

[logv1]; !log of the mean of the burnout residual variance, omega_0;

[logv2]; !log of the mean of the who residual variance, omega_1;

[trend_bu];!mean of trend for burnout, gamma_80;

[trend_wh];!mean of trend for who, gamma_90;

burnout; !burnout intercept variance, tau_00;

who; !who intercept variance, tau_11;

phi1; !burnout autoregressive slope variance, tau_22;

phi2; !who autoregressive slope variance, tau_33;

phi3; !who^ on burnout^1 slope variance, tau_44;

phi4@0.001; !burnout^ on who^1 slope,tau_55;

logv1; !between-person variance of burnout residual variance, tau_66;

logv2; !between-person variance of who residual variance, tau_77;

trend_bu;!between-person variance of burnout trend, tau_88;

trend_wh;!between-person variance of who trend, tau_99;

OUTPUT:

tech1 tech3 tech8 stdyx stand(cluster) cinterval;

PLOT:

TYPE = PLOT3;
